# A single stereogenic center matters: Development of stereodefined anti-tau PMO-gapmers

**DOI:** 10.1016/j.omtn.2025.102519

**Published:** 2025-04-28

**Authors:** Suxiang Chen, Bal Hari Poudel, Rakesh Naduvile Veedu

**Affiliations:** 1 Personalised Medicine Centre, Health Futures Institute, Murdoch University, Murdoch, WA 6150, Australia; 2 Precision Nucleic Acid Therapeutics, Perron Institute for Neurological and Translational Science, Nedlands, WA 6009, Australia; 3 ProGenis Pharmaceuticals Pty., Ltd., Bentley, WA 6102, Australia; 4 SynGenis Pty., Ltd., Bentley, WA 6102, Australia

## Main text

**Figure 1:**
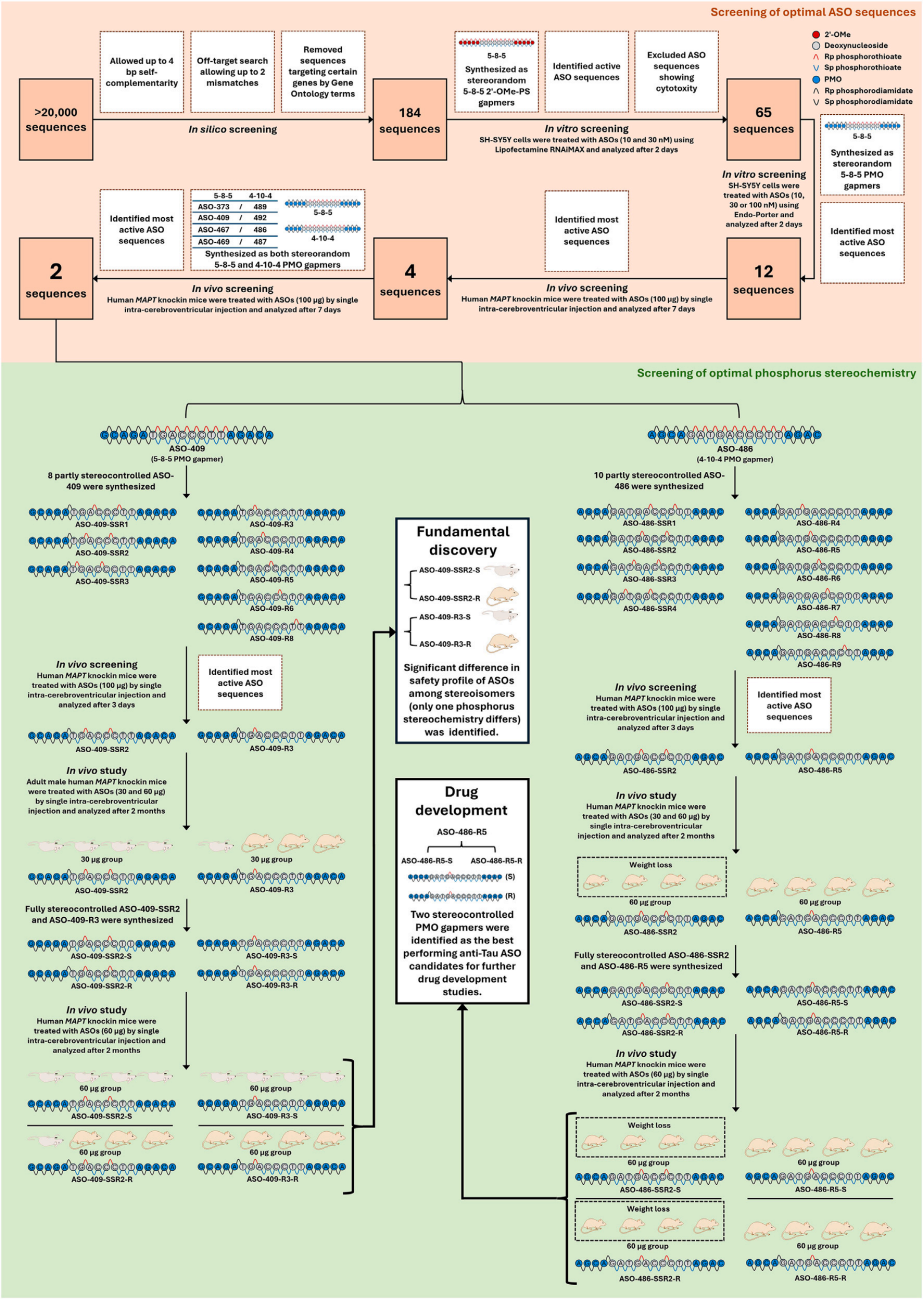
Identification of best-performing stereodefined anti-tau PMO-gapmers and discovery that variation of stereochemistry on a single phosphorous stereogenic center significantly affects the safety profile of ASOs

Antisense oligonucleotides (ASOs) have been utilized for developing RNA-targeting agents that act as an inhibitor of microtubule-associated protein tau (*MAPT*) for the treatment of tauopathies. Although several anti-tau ASO candidates have been reported that could reduce *MAPT* expression either through RNase H-mediated mRNA degradation or splice switching, novel designs of chemically modified ASOs are still needed to improve their activity and safety profile. Moreover, the development of a stereodefined anti-tau ASO is highly desirable due to differences in efficacy and toxicity between diastereomers. Kunihiko Kanatsu et al.^1^ identified two best-performing fully stereocontrolled phosphorodiamidate morpholino oligomer (PMO) gapmers (ASO-486-R5-S and ASO-486-R5-R) targeting *MAPT* mRNA after performing a screening of optimal ASO sequence and subsequent screening of optimal phosphorous stereochemistry (Figure 1). Surprisingly, a dramatic difference in safety profiles between stereoisomers (ASO-409-R3-S versus ASO-409-R3-R, and ASO-409-SSR2-S versus ASO-409-SSR2-R), which only differ in one single phosphorous stereochemistry, was also observed (Figure 1). Fundamentally, this work not only revolutionizes ASO design by adopting PMO as the chemistry of wing regions in a gapmer but also highlights the importance of stereopattern screening in identifying ASO leads since as few as one phosphorous stereogenic center (generating two possible stereoisomers) matters in determining *in vivo* toxicity.

Both gapmer-like ASOs (a DNA gap flanked by two wings constructed with nucleotide analogs such as 2′-O-methyl [2′-OMe], 2′-O-methoxyethyl [2′-MOE], and locked nucleic acid [LNA]) and fully modified ASOs have been employed for inhibiting the expression of target genes through RNase H involvement and steric blocking (e.g., translational repression and splice switching), respectively. However, gapmers are a more preferable option for the purpose of gene knockdown, and nearly all FDA-approved ASO-based gene inhibitors are gapmers (mipomersen, inotersen, eplontersen, tofersen, and olezarsen) except for fomivirsen (a gapmer without wings), which also recruits RNase H. To date, a few gapmers with 2′-MOE- or LNA-modified wings on a full or partial phosphorothioate (PS) backbone have been developed as *MAPT* inhibitors, such as MAPT_Rx_ (ISIS 814907/BIIB080), NIO752, Tau^ASO−12^, and ASO-001933.^2^

PMOs are characterized by their excellent safety profile and resistance to a variety of nucleases. However, PMO was not compatible with other chemistries to synthesize chimeric ASOs (gapmer and mixmer), and therefore, researchers had to explore PMO analogs such as morpholino nucleic acid (MNA) and thiomorpholino oligonucleotide (TMO) for constructing chimeras. On the other hand, PMOs have been widely used as splice-switching ASOs, and so far, all FDA-approved ASO drugs that induce exon skipping are PMOs (eteplirsen, golodirsen, viltolarsen, and casimersen), despite being designed for rescuing dystrophin expression instead of knocking it down.^3^ *MAPT*-specific PMOs that are able to reduce tau protein production through exon-skipping-induced removal of the start codon and/or generation of a premature stop codon, which results in nonsense-mediated mRNA decay (NMD), were identified more than a decade ago.^4^

In this work, for the first time, stereodefined *MAPT*-specific RNase H-dependent PMO-gapmers (DNA-PS gap flanked by PMO wings) were developed through a two-step screening process (Figure 1). Sequence screening was first conducted to identify the best-performing ASO sequence and configuration. Initially, over 20,000 ASO sequences were designed and narrowed down *in silico* by multiple criteria to 184 hit sequences, which were synthesized as stereorandom 5-8-5 2′-OMe-PS gapmers for *in vitro* screening in SH-SY5Y cells. After excluding the ones that exhibited cytotoxicity, 65 active sequences were identified through *in vitro* screening, synthesized as stereorandom 5-8-5 PMO gapmers, and again screened *in vitro* in SH-SY5Y cells. As a result, the 12 most active candidates were selected and entered a 7-day *in vivo* screening in human *MAPT* knockin mice, which led to the identification of the four most active sequences. Two PMO-gapmer configurations (5-8-5 and 4-10-4) of the four sequences were then evaluated *in vivo* (7 days) to find the most efficient ones. ASO-409 (5-8-5 configuration) and ASO-486 (4-10-4 configuration) were identified as the optimal candidates at the end of the sequence screening process.

Screening of optimal phosphorus stereochemistry started with synthesis and subsequent *in vivo* investigation (3 days) of eight and ten different partially stereocontrolled counterparts (including a single *R*p walk and a 3′-*S*p*S*p*R*p-5′ stereotriad^5^ walk in the gap region) of ASO-409 and ASO-486, respectively. As a result, ASO-409-SSR2, ASO-409-R3, ASO-486-SSR2, and ASO-486-R5 were identified as the most active candidates, and their fully stereocontrolled counterparts were then synthesized. In a 2-month *in vivo* study, it was observed that mice treated with ASO-409-SSR2-S and ASO-409-R3-S were all dead within 6 weeks, while only one death was observed in the group treated with ASO-409-SSR2-R (differing from ASO-409-SSR2-S in only one phosphorus stereochemistry). Furthermore, all mice in the ASO-409-R3-R (differing from ASO-409-R3-S in only one phosphorus stereochemistry) treatment group did not show any mortality. These results indicate that the difference in a single phosphorus stereochemistry matters in determining the safety profile of ASOs. On the other hand, although mice treated with ASO-486-SSR2-S and -R did not show mortality, they exhibited body weight loss; ASO-486-SSR2-S even caused increased cytokine release in mice. Therefore, although ASO-486-SSR2-S and -R displayed slightly better *in vivo MAPT* inhibitory efficiency than ASO-486-R5-S and -R, they were not selected as lead candidates. On balance, ASO-486-R5-S and -R are not only efficacious but also safe *in vivo*, at least for 2 months. Thus, both ASO-486-R5-S and -R were selected as the final lead candidates in this work.

In summary, on the one hand, this work contributes to the field by offering single-molecule (stereopure) anti-tau PMO gapmers that are capable of efficiently inhibiting *MAPT* expression without toxicity in an *in vivo* mouse model; this is the first report that PMO, one of the most popular chemistries for uniformly modifying splice-switching ASOs, was utilized to construct gapmer-like ASOs that induce RNase H-mediated degradation of target RNA, which greatly expands the application of PMO chemistry. On the other hand, the fundamental discovery made in this work (the safety profile of ASOs could be determined by the difference of a single phosphorous stereochemistry) highlights the necessity of evaluating and identifying stereodefined ASO candidates with defined safety profiles and efficacy in the drug development process in order to avoid concerns about the safety and efficacy of a mixture of ASO diastereomers, which potentially causes batch-to-batch variation in the properties of an ASO drug. However, since the maximum duration of *in vivo* studies conducted in this work was limited to 2 months, in an attempt to ensure sustained safety and efficacy of the identified anti-tau PMO gapmers, long-term *in vivo* study is required in the future. Finally, it can be predicted that the PMO-gapmer construct will be adopted by more laboratories to develop stereodefined RNase H-dependent ASOs that target different diseases.

## Declaration of interests

The authors declare no existing conflicts of interest.
